# Factors associated with antipsychotic use in non-psychotic depressed patients: results from a clinical multicenter survey

**DOI:** 10.1186/s12888-021-03411-y

**Published:** 2022-02-03

**Authors:** Jingjing Zhou, Tong Zhu, Xuequan Zhu, Britta Galling, Le Xiao

**Affiliations:** 1 The National Clinical Research Center for Mental Disorders & Beijing Key Laboratory of Mental Disorders & Beijing Anding Hospital, Capital Medical University, No 5. Ankang Lane, Deshengmen Wai, Xicheng District, Beijing, 100088 China; 2 Advanced Innovation Center for Human Brain Protection, Capital Medical University, Beijing, China; 3 Department of Child and Adolescent Psychiatry and Psychotherapy, Centre for Integrative Psychiatry, School of Medicine, Kiel, Germany; 4 Department of Child and Adolescent Psychiatry, Psychosomatic Medicine and Psychotherapy, Charité-Universitätsmedizin Berlin, Berlin, Germany; 5 Department of Child and Adolescent Psychosomatic Medicine and Psychotherapy, Altona Children’s Hospital, Hamburg, Germany

**Keywords:** Major depressive disorder, Antipsychotics, Antidepressants, Augmentation, Combination

## Abstract

**Background:**

The combination of antipsychotics is not well studied among non-psychotic major depressive disorder (MDD). This study aims to explore the antipsychotics use in this population and its associated factors.

**Methods:**

This cross-sectional and multi-site study was conducted in 11 sites of China. one Thousand five hundred three eligible MDD patients after 8–12 weeks of antidepressant treatment were included consecutively. A structured questionnaire was used to obtain socio-demographic data and medical histories. The Chinese version of the Quick Inventory of Depressive Symptomatology-Self-Report (QIDS-SR), the Patient Health Questionnaire-15 (PHQ-15) and the Sheehan Disability Scale (SDS) were used for patient self-rating. Logistic regression model was used to explore the associated factors that could potentially be influential for the use antipsychotic augmentation.

**Results:**

Overall, quetiapine (43.4%) was the most commonly used as an adjunct to antidepressants, followed by olanzapine (38.8%). And antipsychotics were commonly combined with escitalopram (23.1%), venlafaxine (21.7%), sertraline (14.8%). The factors influencing the combination of antipsychotics in non-psychotic depressed patients included service setting (OR = 0.444; *p* < 0.001; 95%CI = 0.338–0.583), comorbidity of physical illness (OR = 1.704; *p* < 0.001; 95%CI = 1.274–2.278), PHQ level (OR = 0.680; *p* < 0.001; 95%CI = 0.548–0.844), SDS level (OR = 1.627; *p* < 0.001; 95%CI = 1.371–1.930) and antidepressants co-treatment (OR = 2.606; *p* < 0.001; 95%CI = 1.949–3.485).

**Conclusions:**

Antipsychotics use is common among non-psychotic MDD patient. Service setting, comorbidity of physical illness, somatic symptoms, social functioning and engagement, and antidepressants co-treatment could be the factors associated with the antipsychotics use in MDD patients.

## Introduction

According to WHO estimates - major depressive disorder (MDD) is the leading cause for disability worldwide, with about 350 million affected people [1]. The lifetime prevalence of MDD in China was 3.4% [2]. Antidepressants are the cornerstone of depression treatment, being recommended as first-line treatment in many MDD guidelines [3–5]. However, antidepressant monotherapy solely may result in incomplete remission of MDD in some patients which consequently lead to chronicity, residual symptoms and treatment resistance [6–9]. Because of the acts on multiple receptor systems of the antipsychotics augmentation, antipsychotics is a recommended strategy for severe episode, inadequate response to monotherapy or treatment resistant MDD [3–5]. Moreover, there is a growing body of evidence that supports the use of atypical antipsychotics as augmentation agents for nonpsychotic MDD [10].

Even though there is some evidence supporting the use of atypical antipsychotics as augmentation strategy in non-psychotic MDD, discussion on the risk-benefit-ratio of this strategy have been controversial [9, 11–15]. The benefits of quetiapine and aripiprazole as augmentation strategy are established for MDD, and they have been approved by the Food and drug administration as adjunctive treatment for MDD [16]. In terms of pharmacological mechanism, both antipsychotics have blocking effect on 5-hydroxytryptamine (5-HT) receptors, improving anxiety, depression, attention and kinetic energy [9]. However, previous studies found that the interaction of antidepressants and antipsychotics increases the risk of adverse events [15]. Therefore, as the synergist of antidepressant treatment, antipsychotics use in the treatment of MDD still need further systematic research [9].

It is of interest to examine whether MDD patients benefit from antipsychotic augmentation strategies in clinical setting. In view of very limited empirical research on regarding the associated factors of antipsychotics use in MDD patients. In view of very limited empirical research on regarding the associated factors of antipsychotics use in MDD patients, and the findings has never been examined in a real-world setting. Therefore, the aims of the study were to investigate the rate of antipsychotics augmentation with antidepressants in non-psychotic depressed patients in clinical settings; and to explore the factors associated with antipsychotics augmentation.

## Methods

### Participants and setting

This study was part of a cross-sectional, multi-site survey on the clinical features of MDD patients after 8–12 weeks of antidepressant treatment with or without antipsychotics (clinicians’ choice). The detail of study was reported elsewhere [17].

A total of 1503 MDD patients were enrolled in this study from September 2014 to July 2015 at 11 sites including psychiatric hospitals and general hospitals in Beijing, Shanghai, Guangzhou, Shenzhen, Nanjing, Xi’an, Shijiazhuang, and Harbin. The study was approved by the Ethics Committee of respective participating hospital. All subjects provided written informed consent to participate in the study.

### Data collection

Basic socio-demographic, clinical characteristics, and service setting were collected using a data collection form designed for this study. Details on the use of psychotropics were retrospectively recorded during the past 8–12 weeks. Adjunctive use of antipsychotics was defined as simultaneous use of antipsychotics with antidepressants during past 8–12 weeks.

Depressive symptoms were measured using the Chinese version of the Quick Inventory of Depressive Symptomatology-Self-Report (QIDS-SR) [18, 19]. The Chinese version of the Patient Health Questionnaire-15 (PHQ-15) [20, 21] was used to measure somatic symptoms with a total score ranges from 0 to 30. The Sheehan Disability Scale (SDS) [22, 23] was used to assess social functioning and engagement, including work/school, social life/leisure activity and family life/home responsibilities.

### Statistical analyses

Data were analyzed using the Statistics Analysis System (SAS 9.4). In univariate analyses, independent samples t-tests, Chi-square test, Fisher’s exact test and Wilcoxon test were used as appropriate. Logistic regression model was used to measure the independent contribution of factors that could potentially be influential for the decision to use antipsychotic augmentation, including all factors that were significant between the antipsychotic and non- antipsychotic groups or considered clinically significant, such as basic demographic and illness characteristics and treatment characteristics (antidepressants cotreatment, switch antidepressant, concomitant benzodiazepine and non-benzodiazepine). Stepwise method was used for variables screen (enter standard:*p* < 0.05, elimination standard:*p* < 0.10). The statistical significance for all tests was set at *p* < 0.05 (two-sided).

## Results

### Demographic characteristics of the patients

A total of 1503 patients with non-psychotic MDD were analyzed in this study. The mean age was 43.8 and 62.8% were female. 65.3% of MDD patients were in their first-episode. 334 (22.2%) patients were treated with antipsychotics augmentation. There was no statistically significant difference between patients with antipsychotics and patients without antipsychotics in demographic features (Table 1).

**Table 1:** Comparison of subjects treated concomitant with or without an antipsychotic

Factors	Total sample (*n* = 1503)	With AP (*n* = 334)	Without AP (*n* = 1169)	t/*χ*^*2*^	*P*
Age (year) (%)					
Early adult (age 18–44)	834(55.5)	179 (53.6)	655 (56.0)	0.294	0.524
Middle adult (age 45–59)	424(28.2)	94 (28.1)	330 (28.2)		
Late adult (age ≥ 60)	245(16.3)	61 (18.3)	184 (15.7)		
Gender female (%)	944(62.8)	212 (63.5)	732 (62.6)	0.081	0.775
First-episode patient (%)	982(65.3)	205 (61.4)	777 (66.5)	0.972	0.085
Service setting (%)					
Psychiatric	730(48.6)	111 (33.2)	619 (53.0)	40.433	**< 0.001**
General medical	773(51.4)	223 (66.8)	550 (47.0)		
Comorbidity of physical illness (%)	319 (21.2)	99 (29.6)	220 (18.8)	18.194	**< 0.001**
Family history (%)	157 (10.5)	38 (11.4)	119(10.2)	0.398	0.528
QIDS-SR total score (%)					
0–5	770(51.2)	168 (50.3)	602 (51.5)	12.718	**0.002**
6–10	477(31.7)	89 (26.6)	388 (33.2)		
≥ 11	256(17.0)	77 (23.1)	179 (15.3)		
PHQ-15 total score (%)					
0–5	921(61.3)	228 (68.3)	693 (59.3)	10.817	**< 0.005**
6–10	414(27.5)	69 (20.7)	345 (29.5)		
≥ 11	168(11.2)	37 (11.1)	131 (11.2)		
SDS total score (%)					
0–5	771 (51.3)	164 (49.1)	607 (51.9)	42.147	**< 0.001**
6–10	358 (23.8)	47 (14.1)	311 (26.6)		
> =11	374 (34.9)	123 (36.8)	251 (21.5)		

*QIDS-SR* Quick Inventory of Depressive Symptomatology-Self-Report, *PHQ-15* Patient Health Questionnaire-15, *SDS* Sheehan Disability Scale

### Comparison of patients with antipsychotics and patients without antipsychotics

There was a significant difference in the use of antipsychotic between general hospitals and psychiatry special hospitals (66.8% vs. 33.2%, *p* < 0.001). The severity of MDD (*p* = 0.002), physical comorbidity (*p* < 0.001) and physical symptoms (*p* < 0.005), and poor social function (*p* < 0.001) were significantly different between the patients using antipsychotics and the patients not using antipsychotics (Table 1).

### Types of antipsychotics in augmentation with antidepressants

Among 334 patients with antipsychotics, dozens of patients had been treated with more than one antipsychotic during past 8–12 weeks. As shown in Table 2 of the list of antipsychotics, quetiapine (*n* = 168, 43.4%) was the most commonly used as adjunctive antipsychotic therapy, followed by olanzapine (*n* = 150, 38.8%).

**Table 2:** Types of antipsychotics concomitant with antidepressants in patients

Antipsychotics	Number of use	Rate of use, %
**Quetiapine**	168	43.4
Olanzapine	150	38.8
Aripiprazole	24	6.2
Risperidone	11	2.8
Clozapine	4	1.0
Ziprasidone	3	0.8
Amisulpride	2	0.5
Sulpride	20	5.2
Perphenazine	5	1.3
**Total**	387	–

### Types of antidepressants used in MDD patients

Numbers of subjects had been treated with more than one antidepressant and antipsychotics during past 8–12 weeks. The most frequently used antidepressants were escitalopram (*n* = 439, 24.0%), venlafaxine (*n* = 417, 22.8%) and sertraline (*n* = 226, 12.4%) (Table 3). Antipsychotics were commonly combined with escitalopram (23.1%), venlafaxine (21.7%) and sertraline (14.8%).

**Table 3:** Types of antidepressants used in patients

Antidepressants	Number of use	Rate of use (%)	Number in adjunct AP	Rate in adjunct AP (%)
Escitalopram	439	24.0	97	23.1
Venlafaxine	417	22.8	91	21.7
Sertraline	226	12.4	62	14.8
Paroxetine	152	8.3	26	6.2
Duloxetine	195	10.7	53	12.6
Fluoxetine	93	5.1	26	6.2
Citalopram	93	5.1	14	3.3
Mirtazapine	195	10.7	46	11.0
Trazodone	19	1.0	5	1.2
**Total**	1829	–	420	–

*AD* antidepressants, *AP* antipsychotics

### Factors associated with the use of antipsychotics in multivariate analyses

The dependent variables included age, first episode, the duration of illness, service setting, comorbidity of physical illness, PHQ level, QIDS level, SDS level and antidepressants co-treatment. Logistic regression analyses revealed service setting (OR = 0.444, 95%CI: 0.338, 0.583), comorbidity of physical illness (OR = 1.704, 95%CI: 1.274, 2.278), PHQ level (OR = 0.680, 95%CI: 0.548, 0.844), SDS level (OR = 1.627, 95%CI: 1.371, 1.930) and antidepressants co-treatment (OR = 2.606, 95%CI: 1.949, 3.485) were significantly associated with the use of antipsychotics (*p* < 0.001) (Table 4).

**Table 4:** Logistic regression modeling of factors associated with use of APs with ADs

Factors	B	*S*_*x*_	*χ*^*2*^	*P*	*OR*	*95%CI*
Service setting(psychiatric)	−0.812	0.139	34.138	< 0.001	0.444	0.338, 0.583
Comorbidity of physical illness	0.533	0.148	12.924	< 0.001	1.704	1.274, 2.278
PHQ level	− 0.386	0.110	12.221	< 0.001	0.680	0.548, 0.844
SDS level	0.487	0.087	31.147	< 0.001	1.627	1.371, 1.930
Antidepressants co-treatment	0.958	0.148	41.724	< 0.001	2.606	1.949, 3.485

*PHQ-15* Patient Health Questionnaire-15, *SDS* Sheehan Disability Scale

## Discussion

This study explored the prescribing patterns of antipsychotics in the treatment of non-psychotic MDD in China. Our finding confirms that antipsychotics use (22.2%) was common among non-psychotic MDD patient. Quetiapine was the most commonly used in depressed patients, followed by olanzapine. There is increasing literature supporting the efficacy of add-on quetiapine or olanzapine in the treatment of MDD. Moreover, the FDA of the United States approved olanzapine and quetiapine as adjunctive agents for MDD and the combination of olanzapine with fluoxetine and is also approved for use in treatment-resistant depression [16]. Previous studies have found that quetiapine has a significant synergistic effect on antidepressants [24] and the efficacy of escitalopram and quetiapine was comparable in the treatment of MDD [25]. Moreover, escitalopram and quetiapine combination therapy reduced hypothalamic–pituitary–adrenocortical (HPA) activity, and the inhibitory effect of HPA may be the mechanism of antidepressant effects [26].

In this study, we found that antidepressants co-treatment is the leading factor associated with antipsychotics use. The guideline states that when the replacement of antidepressants is ineffective, the combination of two antidepressants with different mechanisms of action could be adopted. Approximately one third of MDD patients achieve symptom remission after antidepressant monotherapy, and switching to different antidepressant brings the cumulative remission rate to 50–55% [27]. Antidepressants co-treatment is a useful treatment option for treatment-resistant depression [28], and antipsychotics augmentation also improve the remission rate of treatment-resistant depression [27, 29, 30]. Therefore, antidepressants co-treatment often indicates treatment-resistant, which could be an associated factor with the adjunctive use of antipsychotics.

Function impairment is also associated with the severity of depression [4, 5, 31], which may lead to antipsychotics use. However, the relationship of antipsychotics uses with improving social function of depressed patients need to be studied in future follow-up studies.

Somatic symptoms and the severity of depression affect the combination of antipsychotics [32, 33]. With the standardization of treatment, many depressed patients with somatic symptoms consider using serotonin noradrenergic reuptake inhibitors (SNRIs), which have been used in the pain of diabetic neuropathy and painful physical symptoms (backaches, headaches, muscle aches) of depression. The management of pain is achieved through the noradrenergic pathway addressing the peripheral afferent nociception fibers and the rostroventromedial medulla addressed by the 5-HT pathways traversing the neo- and paleospinothalamic tracts [34, 35]. In this study, we cannot make a conclusion about the causal relationship between the severity of depression and antipsychotics use. We speculate that Antipsychotics use in this study may improve depressive symptoms. Future prospective studies should be carried out to explore the causal relationship.

Finally, we found service setting is a crucial influencing factor of antipsychotics use. General hospitals are more likely to use antipsychotics than psychiatry special hospitals. In China’s medical system, psychiatric hospitals have more alternative therapies such as modified convulsively electroconvulsive therapy (MECT), repetitive transcranial magnetic stimulation (rTMS), psychotherapy, while in most psychiatric outpatients clinics of general hospitals only drug therapy is available. Therefore, we presume that clinicians in general hospitals preferred antipsychotics augmentation in improving not only depressive symptoms but also somatic symptoms. Although psychiatric specialized hospitals are more likely to manage more challenging patients for whom augmentation strategies are more likely needed. Psychiatric specialized hospitals are professional in the management of depression, including both clinical protocol and infrastructure support compare with general medical [36]. Availability of alternative treatments in psychiatric specialized hospitals could reduce antipsychotics use. In addition, clinicians in psychiatric hospital maybe more cautious about the side effects of antipsychotics [11, 12, 15]. Moreover, the adverse effects of antipsychotics greatly affect the treatment compliance [9, 13, 37].

## Limitation

There are several limitations in this study. Firstly, this study is a cross-sectional study, which cannot unequivocally establish causal relationship of antipsychotics use and the associated factors. Thus, prospective studies are needed for better assessment of implications of the associated factors with antipsychotics use. Secondly, this study is a secondary analysis of our previously reported study, and the depressed patients included were all responders with ≥50% symptom reduction determined by the visual analogue scale (VAS) [17]. Therefore, the use of antipsychotics in the patients who did not respond to treatment is unknown. Thirdly, the effect of dosage of antipsychotics and antidepressants was not considered. The dose-efficacy relationship is common in the use of drugs, that is, high dose is more effective than low dose. Therefore, low dose of antidepressants is more likely to be combined with antipsychotics. Finally, this study was unable to address the important question of whether augmentation treatment was more effective or less well tolerated than antidepressant monotherapy.

## Data Availability

The data analyzed in this study is subject to the following licenses/restrictions: These data are provided for the purpose of statistical reporting and analysis only. Requests to access these datasets should be directed to corresponding author, Le Xiao, xiaole@ccmu.edu.cn.

## Abbreviations

MDD: Major depressive disorder
QIDS-SR: The Quick Inventory of Depressive Symptomatology-Self-Report
PHQ-15: The Patient Health Questionnaire-15
SDS: The Sheehan Disability Scale
HPA: Hypothalamic–pituitary–adrenocortical
5-HT: 5-hydroxytryptamine
MECT: Modified convulsively electroconvulsive therapy
rTMS: Repetitive transcranial magnetic stimulation
